# The Role of Polycomb Repressive Complex in Malignant Peripheral Nerve Sheath Tumor

**DOI:** 10.3390/genes11030287

**Published:** 2020-03-09

**Authors:** Xiyuan Zhang, Béga Murray, George Mo, Jack F. Shern

**Affiliations:** 1 Pediatric Oncology Branch, Tumor Evolution and Genomics Section, Center for Cancer Research, National Cancer Institute, National Institutes of Health, Bethesda, MD 20892, USA; xiyuan.zhang@nih.gov (X.Z.); bega.murray@nih.gov (B.M.); george.mo@nih.gov (G.M.); 2 The Patrick G Johnston Centre for Cancer Research, Queen’s University Belfast, 97 Lisburn road, Belfast BT9 7AE, UK; 3 SUNY Downstate Health Sciences University, Brooklyn, NY 11203, USA

**Keywords:** neurofibromatosis, malignant peripheral nerve sheath tumor, MPNST, polycomb repressive complex, PRC2

## Abstract

Malignant peripheral nerve sheath tumors (MPNSTs) are aggressive soft tissue sarcomas that can arise most frequently in patients with neurofibromatosis type 1 (NF1). Despite an increasing understanding of the molecular mechanisms that underlie these tumors, there remains limited therapeutic options for this aggressive disease. One potentially critical finding is that a significant proportion of MPNSTs exhibit recurrent mutations in the genes *EED* or *SUZ12,* which are key components of the polycomb repressive complex 2 (PRC2). Tumors harboring these genetic lesions lose the marker of transcriptional repression, trimethylation of lysine residue 27 on histone H3 (H3K27me3) and have dysregulated oncogenic signaling. Given the recurrence of PRC2 alterations, intensive research efforts are now underway with a focus on detailing the epigenetic and transcriptomic consequences of PRC2 loss as well as development of novel therapeutic strategies for targeting these lesions. In this review article, we will summarize the recent findings of PRC2 in MPNST tumorigenesis, including highlighting the functions of PRC2 in normal Schwann cell development and nerve injury repair, as well as provide commentary on the potential therapeutic vulnerabilities of a PRC2 deficient tumor cell.

## 1. Introduction

Neurofibromatosis type 1 (NF1) is a common autosomal dominant disorder caused by inactivating mutations in the tumor suppressor gene *NF1* and affects roughly 1/3000 newborns worldwide [1,2]. The gene *NF1* encodes the GTPase-activating protein neurofibromin (also called neurofibromatosis-related protein) that is a negative regulator of the RAS signaling pathway. Both heterozygous and biallelic loss-of-function (LOF) mutations in *NF1* are associated with hyper-activation of RAS signaling and its downstream targets [3,4,5]. Patients with NF1 are diagnosed when they exhibit two or more of the following symptoms: Six or more café-au-lait macules, two or more neurofibromas or one plexiform neurofibroma (PN), freckling in the axillary or inguinal regions, optic glioma, two or more Lisch nodules, bony dysplasia, or first degree relative with NF1 [6,7,8]. A life-threatening complication of NF1 is an increased risk of the development of the aggressive and highly metastatic soft tissue sarcoma, malignant peripheral nerve sheath tumor (MPNST) [9]. Patients with NF1 have a risk of developing MPNST that is 1000-fold higher than the general population [10,11]. Currently, there are no effective treatments for MPNST other than complete surgical resection with wide negative margins. There are three types of MPNST: NF1-associated, sporadic, and radiation-related, accounting for 50%, 40%, and 10% of all MPNSTs, respectively [12]. Mutations in *NF1* are found in nearly 90% of MPNSTs and frequently involve biallelic loss of the entire gene [13]. As an important tumor suppressor gene, *NF1* is mutated in 8% of all 10,967 The Cancer Genome Atlas (TCGA) curated samples. Interestingly, mutations in *NF1* are not enriched in its GTPase-activating protein domain; rather, they favor missense or truncating lesions that lead to hyper activated RAS signaling.

**Figure 1:**
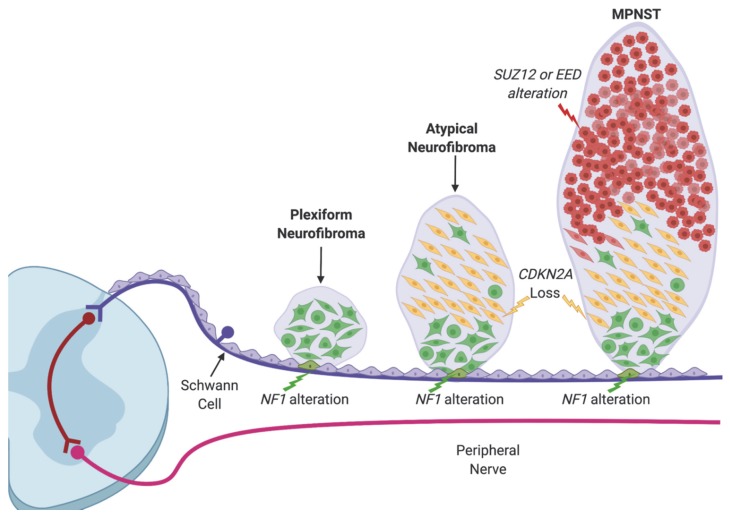
The clinical spectrum and genetic model of nerve tumor development in neurofibromatosis type 1 (NF1). Cells shown here are Schwann cells in the dorsal ganglia root and are affected by the sequential mutations driving the malignant transformation. Green: *NF1* alteration, yellow: *CDKN2A* alteration, and red: PRC2 alteration.

Comprehensive genomic and clinical efforts led to the proposal that there are at least three steps required for cellular transformation during the development of MPNST. These steps are outlined in a genetic model for the development of MPNSTs (Figure 1): 1) fifty percent of NF1 patients will suffer from histologically benign PNs that are caused by the biallelic LOF in *NF1* and associated hyperactivation of RAS signaling [14]; 2) atypical neurofibromas (ANFs, here encompassing distinct nodular lesions and atypical neurofibromatous neoplasms of uncertain biologic potential, ANNUBP) arise within PNs and in addition to hyperactivation of RAS, exhibit heterozygous loss of the genomic locus encompassing the gene *CDKN2A* [15,16]; and 3) approximately 8%-13% of NF1 patients will ultimately have their tumors transform into MPNSTs [11,17], where recurrent mutations in *SUZ12* and/or *EED*, two key components of the polycomb repressive complex 2 (PRC2), lead to loss of tri-methylation of histone H3 lysine 27 (H3K27me3) and de-repression of its target genes [18,19]. Undoubtably, this model oversimplifies the genetic progression of the cell towards malignancy and minimizes the contribution of mutations in other genes, such as *TP53*. However, given the high recurrence of PRC2 alterations, this review will focus on summarizing the current understanding of PRC2 loss in MPNST pathogenesis. 

## 2. Recurrent Mutations in *EED* and *SUZ12* in MPNST 

**Figure 2:**
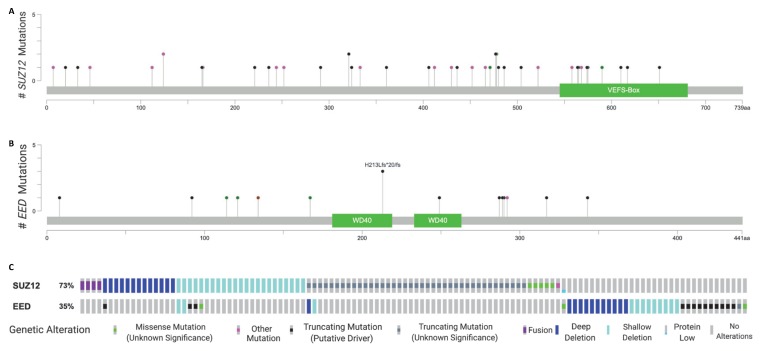
A compiled overview of *SUZ12* and *EED* alterations identified in malignant peripheral nerve sheath tumors (MPNST) published to date, accounting for approximately 75% of sequenced cases. (**A**) A representation of single nucleotide variants (SNVs) discovered in *SUZ12* thus far in MPNST sequencing studies. (**B**) *EED* SNVs identified through sequencing studies. (**C**) An Oncoprint map of the various mutations discovered in both *SUZ12* and *EED* across the MPNST samples sequenced thus far. Figure generated using OncoPrinter and MutationMapper from https://www.cbioportal.org/visualize.

A critical advance in the understanding of the molecular pathogenesis of MPNSTs came from comprehensive genomic analyses of MPNST patient samples through next generation sequencing (NGS). These studies discovered recurrent and frequently mutually exclusive alterations in Embryonic Ectoderm Development *(EED)* and Suppressor of Zeste 12 Protein Homolog (*SUZ12)*, two key components of PRC2, in MPNSTs. Three independent groups nearly simultaneously reported their findings of the genetic aberrations using archived patient MPNSTs [18,19,20]. Lee and colleagues performed whole exome sequencing (WES) of a discovery cohort consisting of 15 MPNSTs and identified five *EED* mutations including four frame-shift and one splice-site alterations, which were associated with loss of heterozygosity, either as a result of deletion of the normal allele or copy-neutral loss and seven *SUZ12* mutations comprised of two homozygous deletions (hom) and six heterozygous loss (het) of one allele [18]. Intriguingly, analysis of WES coupled with whole transcriptome sequencing (RNAseq) of the six MPNSTs with *SUZ12* het loss revealed that two appeared to express the full length of the transcript, with the other 4 exhibiting exonic structural variations (SV). Strikingly, these 4 MPNST samples designated as “het+SV” are all radiation-related, indicating the possibility of local genomic rearrangement caused by previous exposure to radiation in those patients. In the same study, the authors utilized a targeted capture sequencing approach in an additional 37 MPNST samples that were formalin-fixed paraffin-embedded (FFPE) and validated the recurrent mutations in polycomb genes. In another study, Zhang and colleagues performed whole-genome sequencing (WGS) or WES on eight frozen MPNST samples and identified mutations in *EED* or *SUZ12* in 75% of the cases [20]. In their validation cohort of 42 FFPE MPNST specimens, they identified 11 tumors harboring a *SUZ12* mutation (26%). The authors attributed the low fraction of this cohort harboring genetic aberrations in polycomb genes to the partially degraded DNA and false negative rate of the targeted sequencing approach. Finally, DeRaedt et al. used a targeted sequencing approach in a cohort of 51 NF1-associated MPNST samples and discovered 19 samples harboring an *NF1* microdeletion, in which *SUZ12* was frequently co-deleted due to the proximity of these genes in the human genome [19]. More than 50% of the non-microdeletion tumors (32 cases) exhibited inactivating mutations in *SUZ12* or *EED*. Two additional studies verifying the mutation of these polycomb genes were published in 2017. Sohier et al. performed WES in eight NF1-associated MPNSTs and identified *SUZ12* mutations in seven samples and *EED* mutations in two samples [21]. Brohl et al. performed WES on 12 MPNSTs and found genetic alterations in *SUZ12* and/or *EED* in seven samples [13]. The authors also summarized the overall mutational frequency of all five studies that used NGS: *SUZ12* (56.1%) and *EED* (32.5%). Further, MPNST was included in the 2018 soft tissue sarcoma characterization study of TCGA, in which two of the included samples were identified as *SUZ12* mutants in the publicly available data, although no comment was made on these findings in the publication [22]. In summary, a variety of aberrations of the *SUZ12* gene have been identified, including indels, truncating mutations, and missense variants, all of which likely result in aberrant production of this core polycomb protein. Similarly, *EED* is frequently altered through copy number variations in MPNST, as well as through other various LOF mutations including truncating, frameshift, and missense mutations, which lead to abnormal protein production. The *SUZ12* and *EED* mutations identified thus far do not appear to cluster at any known conserved domains within either gene, such as the VEFS binding domain of *SUZ12* or the WD40 protein interaction domain of *EED,* but this may be due to the limited number of MPNST samples that have been characterized for their PRC2 mutant status. Using OncoPrinter and MutationMapper [23,24], we performed meta-analysis of all currently published genome sequencing results of MPNSTs and summarized the accumulated observations of *EED* or *SUZ12* alterations (Figure 2, Table S1).

As another core component of PRC2, Enhancer of Zeste Homolog 2 *(EZH2)* has been implicated as an oncogenic driver in a variety of cancers, playing diverse roles in aiding the development and progression of malignancy [25]. In MPNSTs, however, *EZH2* has recurrently been identified intact, despite the high rate of mutation in other key PRC2 components, *SUZ12* and *EED*. Wassef and colleagues revealed that only the combined loss of both EZH1 and EZH2 in immortalized plexiform neurofibroma-derived cells produced deregulatory effects similar to that observed with the sole absence of either EED or SUZ12 [26]. They also noted a lack of independent function of *EZH2* in the presence of either a *SUZ12* or *EED* mutation, and therefore concluded that the functional redundancy of EZH1 and EZH2 was a contributing factor to the lack of mutation in this component of the PRC2 core.

As noted previously, genetic alterations involving PRC2 and its product H3K27me3 have been reported in a variety of cancer types and most of these studies implicate the EZH2 methyltransferase as the driver of oncogenesis. In contrast to the LOF mutations observed in the *SUZ12* and *EED* subunits of the PRC2 complex, *EZH2* is noted to contribute to tumorigenesis through both over-expression as well as gain-of-function (GOF) mutations [27,28,29,30,31]. A prominent example of an oncogenic GOF mutation of this gene is in human B-cell lymphomas. In this tumor type, a mutation in the SET domain of EZH2 (Tyr641) was present in 21.7% of diffuse large B-cell lymphoma and 7.2% of follicular lymphomas of the germinal-center origin [32]. Subsequent biochemical studies showed that mutant Tyr641, through coordination with the wildtype EZH2, exhibited GOF activities by increasing global H3K27me3 levels [27,33]. Additionally, it was noted that this mutation led to a redistribution of the H3K27me3 repressive mark, thereby allowing for transcriptional activation at certain loci and tumorigenesis in rodent B-cell lymphoma and melanoma models [34]. Further GOF mutations have also been identified within this SET domain of EZH2, involving A677G mutant in B-cell lymphoma [35] and A687V mutant in non-Hodgkin lymphoma [36]. In addition to the association of GOF mutations in *EZH2* with oncogenesis, some studies have implicated the overexpression of this gene in cancer development despite an absence of mutations in the coding region, such as in multiple myeloma [37,38,39], prostate, breast, and endometrial cancers [31,40]. In such cases, the mechanism of EZH2 overexpression could be the result of genomic copy number changes or as a result of epigenomic dysregulation.

Mutations in the PRC2 target histone proteins can also be oncogenic through interference with deposition of the repressive methyl group in the presence of wildtype PRC2. This mechanism is most notable in diffuse intrinsic pontine gliomas and pediatric non-brainstem high-grade gliomas, where mutation in histone H3 yields variant, K27M, that binds and mislocalizes PRC2 in addition to inhibiting its function [41,42]. Interestingly, although these brain tumors exhibit global loss of H3K27me3, several genes retain this repressive mark, and PRC2 itself is required for tumor cell proliferation. This discovery ultimately led to the identification of small molecule inhibitor of EZH2 as potential therapy for this deadly cancer [43].

## 3. The Biochemical, Epigenetic, and Transcriptomic Consequences of PRC2 Loss in MPNST

Polycomb proteins are important regulators of chromatin structure during early development. PRC2 is a highly conserved multimeric complex that plays a distinct role in the transcriptional regulatory activity of the genome through repressive methylation of H3K27 of target genes, which is required in order to induce transcriptional silencing [44,45,46,47]. PRC2 methylates H3K27 to different extents, catalyzing the addition of mono- (H3K27me1), di- (H3K27me2), or tri- methyl groups in cell type specific patterns [48,49]. H3K27me3 is the most well characterized form of methylation at this lysine residue, and occupies around 5%-10% of the genome, while the lesser studied H3K27me2 and H3K27me1 are found at about 50%-70% and 5%-10%, respectively [49,50,51]. PRC2 is highly mobile, with around 80% of the nuclear-located complex undergoing continuous diffusion throughout it, while the remaining PRC2 is stably bound to chromatin [52]. While stably bound PRC2 is often located at H3K27me3 sites, it is rarely found at the site of dimethyl H3K27 that marks intragenic regions and is suggested to act as a repressor of inappropriate activation of cell-specific enhancers and promoters [25,26,27,28]. Unlike the di- and tri- methylated states of H3K27, H3K27me1 does not appear to be involved in transcriptional repression and instead has been found in high abundance at transcribed genes [53]. Prior studies have shown that these global methylation patterns at H3K27 are not regulated by active demethylation at these sites, such as by UTX, Jmjd3, or intracellular demethylases [49]. Target genes that are transcriptionally regulated by PRC2 are essential for embryonic development and cell lineage decisions [48]. 

Detailed characterization of the biochemical, epigenetic, and transcriptomic consequences of PRC2 loss in the MPNST cell remains a goal of current research efforts. In the 2014 study, Lee and colleagues identified a correlation between PRC2 mutation and H3K27me3 loss in MPNST samples, through a combined use of NGS for PRC2 characterization and immunohistochemistry (IHC) staining, to identify loss of H3K27me3 [18]. The identification of IHC screening for H3K27me3 as a reliable biomarker of MPNST led to additional studies that utilized H3K27me3 IHC as a diagnostic marker for MPNST [54,55,56,57,58,59,60,61]. Wojcik and colleagues used this screen to select samples of both PRC2 mutant and PRC2 wildtype status for use in proteomic analyses [62]. These proteomic analyses revealed that the loss of PRC2 caused global changes in post-translational modifications of histones, including 1) a substantial decrease in the transcriptionally repressive modification H3K27me3, 2) broad distribution of the repressive marker H3K27me2, 3) no compensatory gain of other repressive markers, for instance H3K9me3 or H4K20me3, and 4) significant increase in active chromatin markers, including H3K27 acetylation (H3K27ac) and H3K36me2 [62]. Furthermore, they identified that the loss of H3K27me3 across the genome led to the occupancy of those histone tails solely by H3K36me2. Though this paper assumes the commonly theorized transcriptional activation function of H3K36me2, some literature indicates that the dimethylation of H3K36 might be transcriptionally repressive and in contrast with the activation trimethylation of H3K36. Early investigations regarding the location and function of H3K36 di- and tri- methylation marks suggested both distinct locations and opposing roles of these two marks in the regulation of the *Drosophila* genome [63]. This was further commented on by Turberfield et al., who used genome-wide profiling of H3K36me2 to indicate the widespread deposition of transcriptional mark throughout the genome, but with a notable absence on bodies of highly transcribed genes and CpG island-associated gene promoters [64]. Although methylation of H3K36 is commonly associated with transcriptional activation, it has been shown to participate in other cellular processes, including alternative splicing, DNA replication, as well as transcriptional repression [65]. Interestingly, methylation of H3K36 and H3K27 seems to occur mutually exclusively, suggesting that the elevation of H3K36me2 may be a compensatory mechanism of transcriptional repression in cases of PRC2 and H3K27me3 loss [66]. 

**Figure 3:**
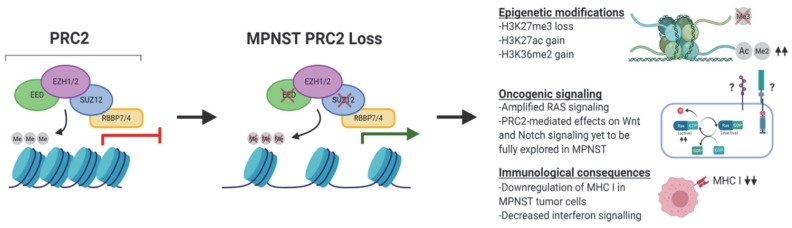
PRC2 structure and consequences of its loss in MPNST. Loss of PRC2 via EED or SUZ12 loss in MPNSTs leads to loss of tri-methylation of histone H3 lysine 27 (H3K27me3) and other potential epigenetic modifications. In addition, PRC2 loss can have a wide variety of consequences on oncogenic signaling and immune surveillance and response.

In addition to the upregulation of recognized PRC2 targets, loss of PRC2 has been implicated in the upregulation of generalized growth and cell division pathways, nucleosome remodeling, and transcriptional activation [18,62]. Somewhat surprisingly, due to the transcriptionally repressive role of PRC2, loss of this complex has also been identified as correlating with the downregulation of certain pathways, including immune-related signaling such as interferon (IFN) signaling and antigen presentation [62]. It is unclear whether these observations are due to alteration of these pathways in the tumor cells or through the reduction of tumor-infiltrating antigen-presenting cells within the tumor. Finally, PRC2 loss was noted to correlate with global DNA hypermethylation at gene promoters and intergenic regions [62]. This DNA hypermethylation was hypothesized by the authors as a potential explanation for the repression of protein expression in the absence of functional PRC2 in MPNSTs. The cooperation of DNA methylation and polycomb complexes in the transcriptional regulation of the genome remains incompletely understood, despite intensive research of the area. It is known that DNA methylation and H3K27me3 mark introduced by PRC2 are typically found in a mutually exclusive pattern across the genome [67,68,69], and the identification of reduced binding capability of PRC2 to nucleosomes with methylated DNA highlights the possibility of an antagonistic relationship between these two epigenetic marks [70,71]. Further, a study by Cooper et al. noted that a decrease in DNA methylation levels corresponded to a redistribution of the H3K27me3 mark across the genome, indicating a role for DNA methylation in polycomb targeting [72]. The effect of polycomb deposited H3K27me3 on DNA methylation levels are less well known, although recent research indicates a role for PRC2 in maintenance of regions of DNA hypomethylation via TET proteins [73]. It remains to be seen whether the loss of PRC2 leads to the upregulation of DNA methylation in human MPNST samples. Despite the wealth of information provided by these research efforts (as summarized in Figure 3), further investigation of the effects of PRC2 loss on the biochemical, epigenetic, and transcriptomic organization of MPNST is crucial to deciphering the mechanisms of tumor development, metastasis, and discovery of potential treatment options for this aggressive disease. 

An unexplored avenue in MPNSTs is the potential relative importance of polycomb repressive complex 1 (PRC1) in a PRC2 deficient cell. PRC1 is a functionally distinct protein complex that plays a critical role in the transcriptional regulation of the genome. PRC1 is responsible for the deposition of mono-ubiquitylation of lysine 119 on histone H2A (H2AK119ub), catalyzed by its E3-ligase subunits, either RING1A or RING1B [74,75]. The mechanism by which PRC1 and PRC2 are recruited to the genome remains an area of debate. It was shown previously that while PRC2 actively methylates H3K27 in target genes and is required in order to induce transcriptional silencing, the Pc subunit of PRC1 also recognizes and binds to this modification, contributing to the transcriptional repression through structural modifications to chromatin, as well as blocking the recruitment of nucleosome remodeling factors such as SWI/SNF [76,77,78,79]. In contrast, the H2AK119ub catalyzed by PRC1 can attract bindings by PRC2, therefore affecting deposition of methylation at H3K27 [72,80,81]. Whether the PRC2 loss in MPNST cells affects the PRC1 complex and the ubiquitination of the epigenome remains to be determined but may represent a unique vulnerability and target in this disease.

## 4. The Role of PRC2 in Schwann Cell Development and Nerve Injury

MPNSTs arise from peripheral nerve branches or fiber sheaths and are thought to be derived from either Schwann cells or pluripotent cells of neural crest origin [82]. In patients with NF1, MPNSTs can arise within the plexiform neurofibromas, and the plexiform neurofibromas grown in the paraspinal region associated with dorsal root ganglia are more likely to go through malignant transformation [83]. In a search for the cells of origin of NF1-associated plexiform neurofibromas, Chen et al. identified a population of GAP43+ PLP+ Schwann cell precursors in the embryonic nerve roots responsible for the neurofibromagenesis [48]. Therefore, understanding the normal development of Schwann cells may be informative for building a model of tumorigenesis of MPNSTs. 

Schwann cells are the primary glial cell of the peripheral nervous system and play a variety of functions including nerve impulse conduction [84], maintenance of the nerve microenvironment [85], presentation of antigens [86], and nerve development and regeneration after injury [85,87]. During embryonic development, the development of peripheral nervous system parallels the development of Schwann cells from neural crest cells through a series of phases starting with migration of neural crest cells and differentiation into Schwann cell precursors, which subsequently become immature Schwann cells. These cells can ultimately differentiate into myelinating and non-myelinating Schwann cells of the mature nerves [88,89,90,91,92]. This highly ordered process of Schwann cell development is tightly regulated by a number of signals, including epigenetic and transcriptional regulations (reviewed in detail [93,94,95]). Notably, in vivo studies showed that disruption of PRC2 or the proper deposit of its product H3K27me3 led to hypermyelination in adult mice [96,97], whilst EZH2 loss in cultured Schwann cells inhibited the myelination process [98]. These inconsistent results may be explained by the differences between in vivo and in vitro systems, and further experiments are needed to resolve these results.

Unlike the ambiguities observed in Schwann cell development, the critical role that PRC2 plays in nerve injury repair is well documented. The Schwann cell injury response involves the reversal of myelin differentiation and downregulation of myelin proteins (reviewed in [99]) and a switch to a repair cell phenotype (reviewed in [87]). In this capacity, the repair Schwann cells can express neurotrophic factors and cytokines that promote neuron survival and axonal regeneration [100,101]. These cytokines recruit macrophages that promote vascularization of distal nerves and assist in the removal of myelin debris that can potentially inhibit axon growth [102,103]. Repair Schwann cells also form tracks known as Bands of Bungner that can guide axon recovery [104].

Nerve injury also induces epigenetic changes in Schwann cells that allows for reprogramming of these cells as they generate the cellular environment required for axon regeneration [105]. PRC2 has been shown to regulate the expression of Schwann cell repair genes and affect nerve injury response via H3K27me3. In this context, PRC2 was found to repress nerve repair genes such as sonic hedgehog (*Shh*), glial-derived neurotrophic factors (*Gdnf*), and brain-derived neurotrophic factors (*Bdnf*) [105]. Nerve injury leads to reversal of PRC2 repression, H3K27 demethylation, and de-repression of these nerve repair genes [106]. Loss of PRC2 repression in an *Eed* conditional knockout mouse model was sufficient to activate these repair genes in uninjured nerves; however, there was no evidence of accelerated nerve injury repair [105]. It is possible that the linkages demonstrating the relationship between nerve injury and PRC2 may yield important clues into the pathogenesis of disease progression of benign neurofibromas to MPNSTs. A critical difference is that PRC2 alteration in nerve injury repair is transient, whereas permanent loss of repression by genetic alteration appears to be needed for malignant transformation. An intriguing result was seen in mice with NF1 deficiencies, where normal mature myelinating Schwann cells exhibited no signs of tumor formation; however, when there was injury to the nerve, neurofibromas developed at those sites [107]. These results and others may indicate that epigenetic programs utilized in the normal process of nerve healing are corrupted by the MPNST cells through the genetic alteration of PRC2.

## 5. Consequences of PRC2 Loss on Oncogenic Signaling in MPNST

Not surprisingly, the consequences of PRC2 loss on the oncogenic signaling within the MPNST cell has become an intensive area of investigation in the NF1 research community. The current paradigm proposes a combination of H3K27me3 loss and de-repression of PRC2 target genes along with other consequential epigenetic alterations in the chromatin landscape promoting oncogenesis [108]. However, it remains unclear exactly what arethe MPNST specific PRC2 target genes that are de-repressed as the malignant transformation takes place. One strategy to answer this question is through transcriptomic and proteomic profiling of human MPNST samples; comparing the PRC2-negative tumors with the PRC2-wild type ones [18,19,62]. These studies have suggested an amplified oncogenic signaling may be playing a role; however, it remains unknown whether these changes are direct or indirect consequences of PRC2 loss. Furthermore, although global increases in active transcription markers H3K27ac and H3K36me3 were observed in PRC2-loss MPNST samples, it remains unclear how these changes affect the three-dimensional structure of the genome and subsequent transcription. Well-controlled model systems, which allow for interrogation of the epigenetic and transcriptomic landscape of MPNST cells, in the PRC2-deficient and intact states would benefit the field tremendously. The resulting cellular signaling changes are hypothesized to contribute to oncogenesis via cell proliferation and growth, however the exact mechanisms that allow this to occur remain unknown. Here, we summarize what is known about the effects PRC2 loss has on RAS, Wnt, and Notch signaling and speculate on the implications that these findings may have for MPNST pathogenesis.

### 5.1. PRC2 Loss and RAS Signaling

As mentioned previously, biallelic LOF in *NF1* is observed in all subtypes of MPNSTs [18,20,109] and this disease can therefore be considered a product of hyperactive RAS signaling pathways. Since MPNSTs can arise within a plexiform neurofibroma, treating these benign tumors is considered a valuable preventive strategy. Indeed, the most effective treatment to date for plexiform neurofibromas is to inhibit the RAS pathway by using MEK inhibitors [110]. However, the loss of *NF1* is necessary but not sufficient for the progression of benign neurofibromas into MPNSTs [111]. Additional genetic mutations either through oncogene amplification or deletions in tumor suppressor genes are required for MPNST transformation [112].

There is evidence that PRC2 loss in MPNSTs contributes to the hyperactive RAS signaling through the epigenetic switch from H3K27me3 to H3K27ac. De Raedt and colleagues found that PRC2 loss amplified NF1 loss-mediated RAS activation and signaling. Using gene set enrichment analysis, this group showed that in SUZ12-depleted cells, there was a significant upregulation of RAS signatures. SUZ12 reconstitution in PRC2-deficient MPNST cells confirmed this result, where downregulation of RAS signatures was noted. Because phospho-ERK levels were unaffected by SUZ12 loss or reconstitution, it was speculated that SUZ12 loss amplified RAS signaling via direct chromatin effects [19]. Evidence for this was seen upon treatment of MPNST cell lines with JQ1, a bromodomain inhibitor, where a similar effect on RAS signatures as SUZ12 reconstitution was noted in the PRC2-deficient cells. Furthermore, the combination of JQ1 and a MEK inhibitor PD-901 was found to cause significant tumor regression in a genetically engineered mouse model with *cis* mutations of *Nf1*, *p53*, and *Suz12* compared to JQ1 or PD-901 alone. The effectiveness of JQ1 in treating PRC2-deficient MPNSTs is consistent with the observation that PRC2-loss triggered increased H3K27ac levels, which is a marker of super enhancers [113]. However, it remains unclear how PRC2 loss alters the global super enhancer landscape and whether additional transcriptional regulators might be involved in the process of malignant transformation. Interestingly, a proteomics-based analysis did not observe specific activation of the RAS pathway in human MPNST samples when comparing tumors with and without intact PRC2 [62]. This inconsistency may be explained by the “contamination” caused by tumor microenvironment when using patient samples or the differences in methodology and requires further investigation.

### 5.2. PRC2 Loss and Wnt Signaling

PRC2 is theorized to suppress Wnt signaling and thereby affect multiple biological processes, such as skeletal muscle differentiation [114], skeletal growth [115], adipogenesis [116], erythropoiesis [117], and intestinal homeostasis [118]. This suppression of Wnt signaling is mediated through a variety of targets within the Wnt pathway, including genes such as Wnt1, Wnt6, Wnt10a, Wnt10b, and Lef1. Therefore, loss of a functional PRC2 repressive complex in MPNST may lead to the upregulation of this signaling pathway, which has previously been identified as a target of oncogenic mutation in many cancer types [119]. Indeed, RNAseq results previously identified enrichment of Wnt signaling in genes significantly upregulated in PRC2-deficient MPNSTs when compared with PRC2-retained samples [18]. Given the active clinical efforts and promising results targeting Wnt signaling, this may represent a tractable therapeutic target in MPNST and supports additional preclinical study and investment. 

Activation of the Wnt pathway has previously been described across several different sarcoma types, including osteo-, Ewing, and rhabdomyosarcomas [120,121,122]. Interestingly, results from an unbiased forward genetic screen highlighted the Wnt signaling pathway as potential driver of oncogenesis in MPNST. In this work, the authors used a *Sleeping Beauty* transposon-based somatic mutagenesis system in mice and found that 17.2% of all genes identified as cooperating with EGFR overexpression were known members of the Wnt/β-catenin pathway [123]. Further, a study by Luscan and colleagues using mRNA expression data and IHC analysis demonstrated altered expression of 20 Wnt genes in MPNST samples compared to benign neurofibromas [124]. These studies provide evidence of Wnt pathway upregulation in MPNST, which could potentially be a direct result of loss of PRC2 in this cancer. The role of PRC2-regulated Wnt signaling has previously been identified in regulating migration and invasion of breast cancer cells, through the regulation of a Wnt signaling pathway inhibitor DKK1 [125], and in multiple myeloma, in which depletion of core PRC2 components EZH1/2 led to overactivation of Wnt signaling [126]. Interestingly, Serresi, Gargiulo, and colleagues have shown that *Eed* deletion cooperated with *Kras* mutant and p53 inactivation to form an invasive mucinous adenocarcinoma [127]. They reported that a chromatin switch between repressive H3K27me3 to its mutually exclusive active mark H3K27ac on the developmental genes of Wnt pathway drove the tumorigenesis. This observation seems to be highly consistent with the genetic alterations reported in MPNSTs. Though the current knowledge of PRC2 regulation of Wnt signaling is limited in the context of carcinogenesis, PRC2 mutant MPNST provides a genetic mechanism and unique model system with which to investigate this interaction further. 

### 5.3. PRC2 Loss and Notch Signaling

Another signaling pathway implicated in MPNST pathogenesis is Notch signaling. The Notch signaling pathway plays a central role in cell differentiation, proliferation, and reprogramming. The Notch family of transmembrane receptors regulate cell fate choices, and aberrant Notch signaling can lead to tumorigenesis in specific cell types such as T-cell lymphomas and pancreatic cancer [128]. While Notch is typically known as a transcriptional activator, several genes have been noted as repressed by Notch activity. The mechanisms as to why this occurs are not fully understood; however, PRC2 may play a role this transcriptional repression.

When Notch receptors are bound and activated, Notch intracellular domains (NICDs) are cleaved and released. NICDs travel to the nucleus and form a ternary complex with the transcriptional coactivator Mastermind (Maml) and DNA-binding transcription factor CSL that can then recruit higher-order transcriptional complexes, resulting in a transcriptional cascade [129,130,131,132]. Han and colleagues found that Notch recruited PRC2 in a Lysine Demethylase 1-dependent manner in T-cell lymphomas, and along with the ternary complex forms a stable transcriptional repressor complex. This leads to enrichment of H3K27me3 repression and loss of H3K4me3 activation, contributing to downstream repressive epigenetic changes [133]. In addition, preliminary data showed that Notch activation led to direct EZH2 and SUZ12 transcriptional induction, although no evidence has shown that they are direct Notch-target genes [133]. Intriguingly, there is also interplay between RAS signaling and Notch signaling; in this manner, Notch signaling seems to be downstream of oncogenic RAS, and wildtype Notch1 is needed for oncogenic RAS-mediated neoplastic transformation of human cells in vitro and in vivo [134].

Although a Notch-mediated PRC2 mechanism has yet to be fully explored in MPNST, Notch signaling may contribute to the malignant transformation of MPNSTs from neurofibromas. Li et al. found that in the sNF96.2 MPNST cells, there was active Notch signaling with NICD generation [135]. Transduction of NICD into rat Schwann cells led to loss of Schwann cell differentiation markers and cellular transformation. These transduced cells had elevated levels of phospho-ERK and Cyclins A, D1, and D2 and were capable of growing into tumor masses when injected into rats. Further research into Notch activation in MPNST is warranted, along with its interplay with PRC2 loss and other driver mutations of MPNST formation.

## 6. Consequences of PRC2 Loss on Tumor Immune Surveillance in MPNST 

Given the growing role of immunotherapy in cancer, there is great interest in understanding the effect that PRC2 loss may play in the ability of MPNST cells to evade immune surveillance. In MPNSTs, PRC2 loss downregulated pathways for antigen presentation and IFN signaling [62]. Proteomic studies revealed decreases in major histocompatibility complex class I (MHC I) expression by tumor cells as well as a decreased infiltration of MPNSTs that lost PRC2 by MHC class II-expressing inflammatory cells. These changes were linked to increased H3K36me2 and H3K27ac as a result of H3K27me3 loss. A proposed mechanism for this observation is PRC2 loss contributing to a decreased IFN signaling as well as the loss of MHC expression. Consistently, restoration of a functional PRC2 or depletion of NSD2 (H3K36me2 methyltransferase) in PRC2-deficient MPNST cell lines resulted in increased MHC I expression and restored IFN pathway expression. It remains unclear whether these changes in immune surveillance and the IFN pathway are directly or indirectly caused by the epigenetic switch of PRC2 loss. Future studies using DNA sequencing coupled with chromatin immunoprecipitation would provide additional mechanistic detail to this observation.

Understanding how to therapeutically modulate the PRC2-induced epigenetic changes in MPNST tumor cells and harness the surrounding immune microenvironment remains a goal of immunotherapeutics. Pilot efforts involving treating PRC2-deficient MPNST cell lines with DNA methyltransferase inhibitors (DNMTi) led to halted cell growth and increased cell death that was associated with increased expression of IFN pathway genes. Additionally, both DNMTi and histone deacetylase inhibitor (HDACi) led to increased MHC I expression in MPNST [62]. Regardless of whether the restoration of IFN pathway genes and MHC I expression is due to direct epigenetic changes, the possibility of using drugs that modulate transcriptional activity opens up the exciting therapeutic possibility of restoring tumor immune surveillance and increasing MPNST targetability.

It is important to note, however, that while PRC2 loss may lead to immune evasion in MPNSTs, in many other cancer types, increased PRC2 activity can actually have a similar effect and also lead to immune surveillance escape through decreased MHC I antigen presentation. A recent study found that PRC2 silenced genes associated with MHC I antigen processing such as MHC I heavy chain genes, the transporter associated with antigen processing (TAP), and the immunoproteasome [136]. In addition, PRC2 restricted transcriptional induction of MHC class I in response to cytokine stimulation in MHC class I deficient tumors such as neuroblastoma and small cell lung cancer. EED or EZH1 and EZH2 inhibition restored expression of MHC I antigen processing genes and effective T cell-mediated immunity in MHC I low cancers. EZH2 inhibition also was shown to enhance tumor immunogenicity through increased interferon signaling, production of proinflammatory chemokines CXCL9 and CXCL10, and modulation of immune cell differentiation.

In cancers where increased PRC2 activity leads to evasion of immune surveillance via decreased MHC I antigen presentation, cell lineage likely plays an important role. Namely, these cells appear to harness embryonic and tissue-specific stem cell programs that are typically regulated by PRC2 to mediate immune evasion [137]. A well-studied example of this phenomenon is in human and mouse melanoma, where PRC2 upregulation was found to be promoted by the presence of tumor-infiltrating T cells [138]. In addition, anti-CTLA-4 or IL-2cx immunotherapy led to increased EZH2, subsequent increases in global H3K27me3, and transcriptional silencing of immunogenicity-related genes including MHC I molecules and antigen processing machinery. In this system, EZH2 inactivation via shRNA or an EZH2 small molecule inhibitor upregulated immunogenicity-associated genes post-immunotherapy downregulation, thus demonstrating immunotherapy-induced gene expression changes that are EZH2-dependent. EZH2 inhibition can synergize with anti-melanoma immunotherapy, stimulating CD8^+^ T cells and suppressing the PD-1/PD-L1 axis. 

Thus, the loss or gain of PRC2 depending on the cancer type can both lead to evasion of immune surveillance via decreased antigen presentation by the tumor cells. It seems likely that PRC2 loss in MPNSTs influences the ability of the immune system to recognize these tumors. One possibility is that MPNSTs arise in the context of NF1-loss mediated hyper activated RAS signaling, which causes decreased interferon signaling and antigen presentation [139,140]. Future work dissecting the interactions of the tumor cell and the host immune system using human samples and immune competent animal models will be required to uncover the mechanistic details of these interactions. Given the potency of immunotherapy in controlling other aggressive metastatic tumor types, this work may have a profound therapeutic impact for patients with MPNST.

## 7. Establishment of Preclinical Modeling of the PRC2 Loss in MPNST as a Pathway to Clinical Translation

Deciphering new vulnerabilities in the MPNST cell that result from PRC2 loss requires the production and characterization of credentialed model systems that faithfully recapitulate human tumors. The most widely used model system in MPNST investigation is that of patient derived tumor cell lines. We summarize here a wide variety of human MPNST cell lines frequently used in preclinical investigations (Table 1). The majority of these cell lines are derived from NF1 patient tumors, and few have been characterized for PRC2 function or SUZ12 expression. This lack of data highlights a potential need for more thorough characterization of these cell lines as we attempt to understand the effects of PRC2 loss in MPNST and its role in oncogenesis. It would also be beneficial to research efforts if *EED* mutant cell lines were identified or developed, which would allow for more complete analyses of the functional consequences of PRC2 complex loss in MPNST. Further, the majority of the commonly used MPNST cell lines were originally obtained from male patient tumors or are of unknown gender origin. This may result in bias of the data obtained from epigenetic research on such MPNST cells, as it has been hypothesized that sex has the potential to affect the epigenetic modification of the nervous system and lead to morphological differences [141,142]. Clarification of the sex of available MPNST cell lines, or the establishment of novel immortalized cell lines from tumors of female patients, may aid the removal of such bias from ongoing MPNST research. Exciting efforts are underway to develop a next generation of model systems, including work by the the NF1 Biospecimen Repository at Johns Hopkins (https://www.hopkinsmedicine.org/kimmel_cancer_center/centers/pediatric_oncology/research_and_clinical_trials/pratilas/nf1_biospecimen_repository.html). Importantly, these efforts include a fully annotated clinical database and biospecimen bank of NF1-associated MPNST primary tumors, cell lines, and novel patient-derived xenografts (PDXs), which are available on request.

A variety of animal models have been used in preclinical investigations of MPNST in an effort to more closely recapitulate the human tumor environment. Murine models are frequently used in this area of research, particularly xenograft or orthograft models involving the engraftment of the human tumor cell lines into mice. Most of the cell lines listed in Table 1 have been utilized in xenograft research of MPNST, including those known to be PRC2 mutant, allowing for biological modelling of tumors possessing this aberration. Another common method of MPNST investigation is the use of PDX models, in which patient tumor is engrafted onto an immunocompromised host, to allow for the investigation of these tumor cells in the context of an in vivo environment. Although there is a growing number of PDX models described [166,167], documentation of PRC2 mutational status has not been routinely commented on. 

Genetically engineered mouse models (GEMMs) are another useful tool for the study of cancer development, progression, and therapeutics, but have proved difficult to produce in the case of PRC2 mutant MPNST. Despite the success of GEMM in contributing to the study of NF1 pathogenesis and plexiform development, the development of MPNST models has been a slow and complex process [168]. An effort was made by De Raedt and colleagues to generate MPNST models through the generation of *Nf1*, *p53,* and *Suz12* mutant mice [19]. They generated *Nf1^+/−^*, *Suz12 ^+/−^* mice in cis, in which the mutant copies of these genes were on a single chromosome, and tumors developed upon spontaneous loss of the wildtype chromosome. This murine model had a high rate of tumor development and decreased survival. They further developed a *Nf1^+/−^*, *Suz12^+/−^*, and *p53^+/−^* cis model, as the p53 tumor suppressor protein is found to be commonly mutated in MPNST. These mutant models were found to have a high rate of spontaneous tumor development, but the tumors were of a wide histological variety, including histiocytic sarcomas, intestinal adenomas, neurofibromas, hepatocellular carcinomas, as well as MPNSTs. While MPNST development was identified in this study, mice frequently succumbed to other cancerous diseases prior to this tumor formation, indicating a lack of efficiency of this MPNST model. Due to the high rate of tumorigenesis and wide variety of tumors as a result of the combination of *NF1, p53,* and *SUZ12* deletion, it is possible that a more effective murine model of MPNST can utilize floxed alleles that conditionally knockout tumor suppressors in the appropriate cells of origin. As mentioned previously, neural crest gives rise to Schwann cell precursors, which subsequently differentiate into immature Schwann cells and then myelinating and non-myelinating Schwann cells after birth [90]. It has been appreciated that the cells of origin that give rise to plexiform neurofibromas and therefore MPNSTs are the Schwann cells in the dorsal ganglia root [83]. Using a genetically engineered *Nf1* floxed, *Cdkn2a/Arf* floxed, and PostnCre mouse model that triggered conditional knockout in the nerve crest derived Schwann cell lineage, Rhodes et al. created one of first models that mimic the human malignant transformation from plexiform to atypical neurofibroma, which eventually developed MPNST with a high penetrance [169]. It is hoped that with the increasingly accurate description of the genetic lesions associated with the tumorigenic formation of human MPNSTs, more MPNST GEMMs will be produced that faithfully recapitulate the genetic lesions and will become available to the researcher community. 

## 8. Conclusions

The advance of NGS enabled the discovery of PRC2 loss in MPNSTs, and the loss of H3K27me3 has become a clinically useful, sensitive, and specific marker for diagnosis. Efforts to understand the consequences of PRC2 loss in MPNST tumorigenesis and to identify novel vulnerabilities in this difficult to treat tumor are areas of intensive focus for both basic and translational researchers. Recent works have discovered that the loss of PRC2 in MPNST likely affects changes in cellular signaling and immune surveillance through alteration of the core epigenetic and transcriptomic landscape in a neuronal specific precursor cell. Further studies will be enabled through a new generation of clinically annotated and genetically profiled patient samples and their derivative MPNST cell lines and PDX models, as well as GEMMs that mimic the clinically observed disease progression from benign plexiform neurofibroma through atypical neurofibroma to MPNST. These and other anticipated advances will hopefully accelerate discovery of mechanistically based strategies for the treatment of this devastating tumor.

## Figures and Tables

**Table 1:** A summary of immortalized cell lines used in MPNST research.

Cell Line	Sex	Synonyms	Origin	PRC2 Status	Ref.
T265	/	T265-2c; T265-2C; T265p21	NF1	Loss [62,143]	[144,145]
90-8	/	MPNST 90-8TL; 90-8TL; NF90-8; NF190-8	NF1	Loss [19]	[146]
ST88-3	M	88-3; NF188-3	NF1	Unknown	[147]
ST88-14	M	ST88.14; ST 88-14; ST-8814; ST8814; 88-14; NF188-14	NF1	Loss [18,19]	[147]
sNF02.2	M	sNF02-2	NF1	WT [148]	[149]
sNF10.1	/		NF1	Loss [150]	[150]
sNF94.3	F		NF1	Loss [150]	[151]
sNF96.2	M	SNF96.2; sNF96-2	NF1	Loss [19,148]	[152]
S462	/		NF1	Loss [19,62]	[153]
S462.TY	/	S462-TY; S462TY	NF1	Unknown	[154]
S520	/		NF1	Unknown	[153]
S805	/		NF1	Unknown	[155]
FMS-1	F		NF1	Unknown	[156]
FU-SFT8710	F		NF1	Unknown	[157]
NFS-1	/		NF1	Unknown	[158]
NMS-2	M		NF1	Unknown	[159]
NMS-2PC	M		NF1	Unknown	[159]
MPNST-14	M		NF1	Unknown	[160]
MPNST642	M		NF1	Unknown	[161]
1507.2	/	S1507-2	NF1	Unknown	[153,162]
STS-26T	/	STS26T; STS26	Sporadic	WT [62,143]	[163]
MPNST-724	/	MPNST724	Sporadic	WT [18]	[160]
HS-Sch-2	F		Sporadic	Unknown	[164]
HS-PSS	M		Sporadic	Unknown	[165]
YST-1	F		Sporadic	Unknown	[165]
FU-SFT9817	F		Sporadic	Unknown	[157]
FU-SFT8611	M		Sporadic	Unknown	[157]

F: Derived from a female patient. M: Derived from a male patient. Loss: Normal function of PRC2 loss determined in the indicated reference. WT: Normal function of PRC2 retained in the indicated reference.

## Supplementary Materials

The following are available online at https://www.mdpi.com/2073-4425/11/3/287/s1, Table S1: Summary of mutations of *SUZ12* and *EED* in human MPNSTs.
